# “ID-ing” the value: how are orthopaedic infectious disease physicians compensated for their time? A national survey

**DOI:** 10.5194/jbji-11-337-2026

**Published:** 2026-06-12

**Authors:** Jessica C. O'Neil, Bethany Lehman, Thorsten Seyler, Nicolas Piuzzi, Sean Ryan, Jessica Seidelman, Julie Reznicek, Poorani Sekar

**Affiliations:** 1 Division of Infectious Diseases, Department of Medicine, University of Pennsylvania, Philadelphia, Pennsylvania, USA; 2 Department of Infectious Diseases, Integrated Hospital Care Institute, Cleveland Clinic Foundation, Cleveland, Ohio, USA; 3 Division of Adult Reconstruction, Department of Orthopaedics, Duke University Hospital, Morrisville, North Carolina, USA; 4 Department of Orthopaedic Surgery, Cleveland Clinic, Cleveland, Ohio, USA; 5 Division of Infectious Diseases, Department of Medicine, Duke University Medical School, Durham, North Carolina, USA; 6 Division of Infectious Diseases, Department of Medicine, Virginia Commonwealth University Health, Richmond, Virginia, USA; 7 Department of Internal Medicine, University of Iowa Carver College of Medicine, Iowa City, Iowa, USA

## Abstract

**Background**: Optimal outcomes in periprosthetic joint infection (PJI) and other complex orthopaedic infections depend on longitudinal, multidisciplinary input from infectious disease (ID) specialists. Much of this work is not directly billable, and the financial structures that support it have not been systematically described in any healthcare system. **Methods**: We distributed an anonymous online cross-sectional survey to ID physician members of the Musculoskeletal Infection Society (MSIS) and the Infectious Diseases Society of America Bone and Joint Interest (IDSA-BOJO) group between 12 December 2025 and 30 January 2026. The survey captured practice setting, clinical and non-clinical orthopaedic ID responsibilities, and sources of compensation and protected time. Data were analysed descriptively; no inferential statistical testing was performed. **Results**: Fifty-two ID physicians responded, 94 % from US academic medical centres. Median ID clinical effort was 70 % (IQR 60 %–81 %); of this, a median of 55 % (IQR 40 %–90 %) was devoted to orthopaedic infections. Compensation was most commonly a base salary plus productivity incentives (53 %) or base salary alone (40 %) and was funded by medicine/ID in 88 % of cases. Care coordination (90 %), curbside consultation (85 %), trainee education (77 %), and guideline development (71 %) were performed; 83 % of respondents received no protected time or dedicated funding for any of these non-billable activities. **Conclusions**: In this descriptive, hypothesis-generating national survey of highly subspecialized US ID physicians, compensation structures were dominated by clinical productivity and rarely included protected time for the non-billable coordination work that underpins modern multidisciplinary PJI care. These findings should be interpreted in the context of a small, academic-predominant sample and of substantial international variation in healthcare financing, and they support the need for larger international studies of how orthopaedic ID expertise is funded and protected.

## 1 Introduction

Periprosthetic joint infection (PJI) is among the most serious complications following total joint arthroplasty (Hewlett et al., 2025). As arthroplasty volumes increase in the United States (US), the burden of PJI continues to rise, intensifying demand for expertise in diagnosis and management (Shichman et al., 2023). Optimal care requires multidisciplinary collaboration across diagnosis, surgical strategy, and antimicrobial therapy. Guidelines emphasize the important role of infectious disease (ID) physicians, whose involvement has been associated with improved outcomes, including reduced hospital length of stay, lower readmission rates, improved treatment success, reduced use of inappropriate antibiotics, and reduced related healthcare costs (Hewlett et al., 2025; Lucas et al., 2025; Ntalos et al., 2019).

The US faces a significant shortage of ID physicians. Approximately 80 % of counties lack an ID physician, with rural areas disproportionately affected (Walensky and McCann, 2025). Workforce concerns have intensified as fellowship recruitment has declined; in the 2024 match, 51 % of ID fellowship positions went unfilled, largely due to compensation disparities relative to other specialties (Andrews et al., 2024). Orthopaedic infection care is particularly vulnerable because it requires sustained collaboration between orthopaedic surgeons and ID specialists, yet the scope of ID participation varies widely across institutions.

The economic impact of PJI further highlights the need for sustainable care models. PJI care can represent a substantial financial burden for health systems, creating financial pressures that often conflict with an expanded need for multidisciplinary infection care and highlighting the importance of reimbursement structures that adequately support ID physicians' involvement (Samuel et al., 2020).

Similar concerns exist internationally. European bone and joint infection networks, including the European Bone and Joint Infection Society (EBJIS), now jointly endorses a PJI definition developed with the European Society of Clinical Microbiology and Infectious Diseases (ESCMID) study group for implant-associated infections (McNally et al., 2021), reflecting the inherent multidisciplinary nature of PJI. Dedicated bone and joint infection units, typified by the Oxford Bone Infection Unit, emphasize the need for protected time for coordination between specialties (Colston and Atkins, 2018). Analogous models in transplant ID, where US programmes provide salary support for non-billable work, and European musculoskeletal-oncology care are built around longitudinal, reimbursed multidisciplinary care (Gronchi et al., 2021; Strauss et al., 2021).

Despite the central role of ID physicians in orthopaedic infection care, little is known about how this work is financially supported in the US. We therefore conducted a national survey of ID physicians with clinical expertise in orthopaedic infections to characterize compensation models, institutional infrastructure and uncompensated responsibilities.

## 2 Methods

### 2.1 Survey design and participants

We developed an anonymous survey (Supplement). Questions assessed employment setting, clinical responsibilities related to orthopaedic infection care and compensation structures. The survey was independently piloted by two ID physicians with expertise in orthopaedic ID care. The survey was distributed electronically via Qualtrics (Provo, UT) to ID physician members of two international professional societies, the Musculoskeletal Infection Society (MSIS) and the Infectious Diseases Society of America Bone and Joint Interest (IDSA-BOJO) group, to intentionally target ID physicians with expertise in orthopaedic infections. Participation was voluntary without incentives. Survey responses were collected between 12 December 2025 and 30 January 2026.

### 2.2 Data analysis

Data were analysed descriptively. Categorical variables are reported as frequencies and proportions. Free-text responses were summarized thematically. No inferential statistical testing was performed given the non-ransom, subspecialty sample and descriptive study design. The study protocol was exempted by the University of Pennsylvania Institutional Review Board.

## 3 Results

**Figure 1:**
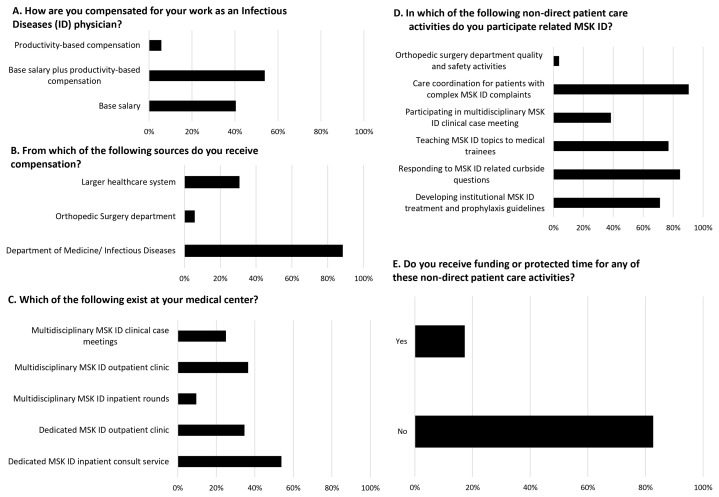
Summary of workforce roles, compensation models, and non-direct patient care activities among surveyed ID physicians. Survey-reported compensation structures, departmental funding sources, availability of orthopaedic ID clinical programmes, involvement in non-direct orthopaedic ID activities, and receipt of protected time among practicing orthopaedic infectious disease physicians.

A total of 52 ID physicians completed the survey. Responses are summarized in Fig. 1.

**Table 1:** Current challenges and proposed interventions in orthopaedic infectious diseases.

Current challenges	Proposed solutions
*Rising clinical burden*: PJI incidence is increasing alongside rising arthroplasty volumes and patient complexity	Shared funding models: implement shared full-time-equivalent support between orthopaedic surgery and medicine/ID divisions
*Inadequate reimbursement*: current reimbursement systems (including CMS) often fail to account for the longitudinal expertise and complex coordination required for successful PJI outcomes	Advocacy for enhanced reimbursement: lobbying for reimbursement mechanisms that adequately support hospitals, surgeons, and ID physicians managing complex infections
*Workforce shortage*: the national shortage of ID physicians is worsening, with over 50 % of fellowship positions remaining unfilled due to low compensation	Formalized compensation: create structured payment models for essential non-billable work, such as multidisciplinary conferences and care coordination
*Uncompensated labour*: 83 % of specialists receive no protected time or pay for non-billable activities like protocol development and OPAT oversight	Proven frameworks: adopt institutional support models used in transplant ID and oncology (tumour boards) to sustain clinical programmes
*Fragmented support*: funding primarily comes from medicine departments (88 %), while support from orthopaedic surgery is rare (6%)	Dedicated resources: invest in dedicated orthopaedic ID nursing, pharmacy resources, and specialized continuing medical education

### 3.1 Practice setting and geography

Most respondents practised in academic medical centres (49/52, 94 %). Respondents reported substantial orthopaedic ID specialization. Median ID clinical effort was 70 % (IQR 60 %–81 %), with a median of 55 % (IQR 40 %–90 %) of that time devoted to orthopaedic infections.

### 3.2 Compensation structure and infrastructure

Base salary with productivity incentives (28/52, 53 %) was the most common compensation model. Funding for orthopaedic ID work primarily came from medicine/ID (46/52, 88 %). Orthopaedic surgery departmental funding was uncommon (3/52, 6 %). Over half of respondents reported a dedicated orthopaedic ID inpatient consult service (28/52, 54 %). Dedicated outpatient orthopaedic ID clinics were present for 18 of 52 (33 %) respondents. Multidisciplinary structures varied: inpatient rounds (9 %), outpatient multidisciplinary clinics (35 %), and case conferences (13/52, 24 %).

### 3.3 Non-direct patient care activities and funding

Most respondents routinely performed care coordination (90 %), curbside consultation (85 %), trainee education (77 %), and guideline development (71 %). Participation in multidisciplinary clinical programmes was reported by 20 respondents (39 %). Most (83 %) received no protected time or funding for non-clinical orthopaedic ID activities. Among the nine who did, support typically came from the Department of Medicine or the larger health system for roles not specific to orthopaedic ID, such as stewardship or education.

## 4 Discussion

In this descriptive national survey, most ID physicians specializing in orthopaedic infections reported no protected time or funding for essential non-billable work central to PJI care. This reflects a mismatch between the complexity of PJI management and compensation models that reward billable encounters, consistent with prior reports that work-relative value-unit assignments underestimate infection-care-related efforts in revision arthroplasty (Samuel et al., 2020; Cortés-Penfield et al., 2024).

Respondents described substantial uncompensated responsibilities, including care coordination, curbside consultation, protocol development, and multidisciplinary discussions, activities that influence surgical planning and patient outcomes. Despite evidence that embedded ID involvement improves clinical outcomes and offsets associated costs, institutional support was limited. Similar patterns have been reported internationally: Denes et al. (2014) prospectively recorded approximately 2700 ID curbside consultations per year at a single French teaching hospital, accounting for 20 % of one physician's clinical time and an estimated EUR 77 000 of non-reimbursed work annually (Denes et al., 2014). Using the Association of American Medical Colleges median academic ID salary as a benchmark, each 0.1 full-time equivalent of unfunded orthopaedic ID effort corresponds to roughly USD 26 000 per physician per year in opportunity cost; although illustrative and not measured in this survey, this estimate reflects the magnitude of uncompensated work reported by respondents.

These gaps may be amplified as arthroplasty volume expands while the ID workforce contracts and fellowship positions remain unfilled. Without deliberate investment in the orthopaedic ID workforce, access to experienced orthopaedic ID specialists may become even more restricted.

Transplant ID offers a potential model: over 75 % of US transplant programmes provide salary support for non-billable multidisciplinary work, recognizing its importance to outcomes and programme viability (Schaenman et al., 2017). Although regulatory and reimbursement mechanisms differ, orthopaedic infection care similarly requires sustained interdisciplinary management of high-risk patients.

Another parallel exists in oncology. Multidisciplinary tumour boards are a standard of care across cancer types and, in the US, are required for accreditation by the American College of Surgeons Commission on Cancer with demonstrated improvements in diagnostic accuracy and outcomes (Specchia et al., 2020). Sarcoma care in particular requires longitudinal multidisciplinary coordination for which European ESMO-EURACAN-GENTURIS guidelines recommend referral to established centres with formalized team structures and protected time for coordination (Gronchi et al., 2021; Strauss et al., 2021; Beird et al., 2022). Given the shared features with PJI, including complex diagnostics, staged treatment, and prolonged follow-up, a similar, reference-centre framework could be considered for complex orthopaedic infections.

Economic considerations further underscore the need for reform. While some tertiary centres achieve financial sustainability for complex PJI referrals, PJIs are widely recognized as a substantial economic burden due to repeat procedures and prolonged hospitalizations (Samuel et al., 2020). Current reimbursement systems often fail to account for the longitudinal ID expertise, emphasizing the need to reassess reimbursement mechanisms, including Centers for Medicare and Medicaid Services policies.

Practical institutional steps (Table 1) could include shared full-time-equivalent support between orthopaedic surgery and medicine divisions, formalized compensation for participation in structured multidisciplinary PJI conferences, dedicated orthopaedic ID nursing and pharmacy resources, and targeted education investments in orthopaedic care.

Key challenges include rising clinical burden, inadequate reimbursement, workforce shortages, uncompensated non-billable work, and fragmented institutional support. Proposed solutions emphasize shared funding models, enhanced reimbursement mechanisms, formalized compensation for essential non-billable activities, adoption of established multidisciplinary care frameworks, and investment in dedicated orthopaedic infectious disease resources.

This study has limitations, including a modest sample size, reliance on self-reported data without objective compensation or productivity metrics, and the absence of inferential statistical testing. MSIS and IDSA-BOJO membership is also weighted toward academic ID physicians with a sustained orthopaedic focus, so the findings cannot be generalized to private-practice, community-hospital, or non-US ID physicians, where reimbursement and workforce structures differ substantially; these settings should be prioritized for future international sampling. Nonetheless, these findings provide an early national characterization of compensation gaps and structural variability in orthopaedic ID practice and should be regarded as hypothesis generating pending confirmation in larger, internationally representative samples.

In conclusion, ID physicians provide essential expertise for PJI and other complex orthopaedic infections, yet in this descriptive US survey, the compensation frameworks reported often did not include dedicated support for the non-billable work central to multidisciplinary PJI care. As arthroplasty volumes rise and the ID workforce remains strained, service-line funding models and structured multidisciplinary support, potentially informed by the transplant-ID and European musculoskeletal-oncology reference-centre frameworks, may help align institutional investment with patient outcomes. Confirmatory international studies with larger, more diverse samples are needed before specific reimbursement policy recommendations can be made.

## Supplement

10.5194/jbji-11-337-2026-supplement The supplement related to this article is available online at https://doi.org/10.5194/jbji-11-337-2026-supplement.

## Data Availability

The data underlying this study are not publicly available at the time of publication in accordance with University of Pennsylvania data sharing and governance policies. The data are available for review upon reasonable request to the corresponding author. Requests for access will be evaluated to ensure appropriate use and adherence to institutional and ethical requirements.
