# Supplementary material for: “ID-ing” the value: how are orthopaedic infectious disease physicians compensated for their time? A national survey
Source: J Bone Jt Infect. 2026 Jun 12;11(3):337–42. doi: 10.5194/jbji-11-337-2026 (PMC13270337; doi:10.5194/jbji-11-337-2026)
Supplement: The supplement related to this article is available online at https://doi.org/10.5194/jbji-11-337-2026-supplement. [file jbji-11-337-2026-supplement.pdf]

Supplement of J. Bone Joint Infect., 11, 337–342, 2026
https://doi.org/10.5194/jbji-11-337-2026-supplement
© Author(s) 2026. CC BY 4.0 License.

JBJI
Journal of Bone and Joint Infection
Open Access

Supplement of

"ID-ing" the value: how are orthopaedic infectious disease physicians compensated for their time? A national survey

Jessica C. O'Neil et al.

Correspondence to: Jessica C. O'Neil (jessica.oneil@pennmedicine.upenn.edu)

The copyright of individual parts of the supplement might differ from the article licence.

Default Question Block

1. What is your primary employment setting? [select one]

- ○ Academic hospital system
- ○ Non-academic hospital system
- ○ Government-based hospital system
- ○ Private practice
- ○ ____________ Other

Do you primarily practice in the United States?

- ○ Yes
- ○ No

In which state do you primarily practice?

[▾]

What percentage of your time is spent practicing clinical ID? (As opposed to other institutional roles such as administrative, research or teaching) [input numeric value between 0-100]

[ ]

What percentage of your clinical ID time is spent caring for patients with musculoskeletal (MSK) ID complaints? (Such as periprosthetic joint infection, fracture related infection, diabetic foot infection, osteomyelitis) [input numeric value between 0-100]

[ ]

5. How are you compensated for your work as an Infectious Diseases (ID) physician? [select one]

- ○ Base salary
- ○ Productivity-based compensation
- ○ Base salary plus productivity-based compensation

From which of the following sources do you receive compensation? [select all that apply]

- ☐ Department of Medicine/ Infectious Diseases
- ☐ Orthopedic Surgery department
- ☐ Other surgical departments
- ☐ The larger healthcare system
- ☐ ____________ Other

Which of the following, if any exist at your medical center? [select all that apply]

- ☐ Dedicated MSK ID inpatient consult service
- ☐ Dedicated MSK ID outpatient clinic
- ☐ Multidisciplinary MSK ID inpatient rounds
- ☐ Multidisciplinary MSK ID outpatient clinic
- ☐ Multidisciplinary MSK ID clinical case meetings

In which of the following non-direct patient care activities do you participate related MSK ID? [select all that apply]

- ☐ Developing institutional MSK ID treatment guidelines
- ☐ Responding to MSK ID related curbside questions
- ☐ Teaching MSK ID topics to medical trainees

- [ ] Participating in multidisciplinary MSK ID clinical case meetings
- [ ] Care coordination for patients with complex MSK ID complaints
- [ ] ______ Other

Do you receive funding or protected time for any of these non-direct patient care activities? [select one]

- ○ Yes
- ○ No

If yes, please describe the source or sources of your funding or protected time. [free text]

Powered by Qualtrics
